# Construction and verification of a prediction model for sleep disorders in older patients with coronary heart disease based on machine learning algorithms

**DOI:** 10.3389/fmed.2026.1834259

**Published:** 2026-06-30

**Authors:** Dali Dong, Xiang Peng, Siling Tan, Hua He

**Affiliations:** 1 Department of Medical Affairs, the Second Affiliated Hospital of Hunan University of Chinese Medicine, Changsha, Hunan, China; 2 School of Nursing, Hunan University of Chinese Medicine, Changsha, Hunan, China; 3 Department of Nursing, The Second Affiliated Hospital of Hunan University of Chinese Medicine, Changsha, Hunan, China

**Keywords:** coronary heart disease, elderly, machine learning, predictive model, random forest, sleep disorders

## Abstract

**Objective:**

To construct and validate a predictive model for sleep disorders in older adults with coronary heart disease (CHD) using machine learning algorithms.

**Methods:**

A cross-sectional survey design was employed to collect clinical data from 338 older adult patients with CHD who were admitted to our hospital between January 2023 and December 2024. Participants were divided into two groups based on the presence of sleep disorders (PSQI > 7). Data were collected from questionnaires and electronic medical records, and subjects were randomly divided into training (241 cases) and validation (97 cases) sets in a 7:3 ratio. Least absolute shrinkage and selection operator (LASSO) regression was used to screen risk factors from the training set, and Logistic regression, random forest (RF), and gradient boosting machine (GBM) models were built. Receiver operating characteristic (ROC) and calibration curves evaluated model performance, with a temporal validation cohort of 152 older CHD patients from our hospital between January and October 2025.

**Results:**

Among 338 patients, 82 (24.26%) had sleep disorders, and 58 (24.07%) in the training group.. In the training set, LASSO identified five risk factors: sex, duration of CHD, chronic gastritis, anxiety, and depression. The three models showed good predictive performance with different strengths. In the training set, the RF model achieved the highest AUC (0.839), accuracy (0.766), and F1 score (0.818); both RF and Logistic regression achieved the highest sensitivity (0.812). In the validation set, the GBM model achieved the highest AUC (0.838) and accuracy (0.733); the Logistic regression model demonstrated the best specificity (0.875) and F1 score (0.825). External validation of the Logistic regression model showed an AUC of 0.785 (95% CI: 0.694–0.876), sensitivity of 0.798, and specificity of 0.658, with calibration curves showing good consistency.

**Conclusion:**

The Logistic regression, GBM, and RF models based on sex, duration of CHD, chronic gastritis, anxiety, and depression all demonstrated good predictive performance.

## Introduction

Coronary heart disease (CHD) is one of the leading causes of disability and mortality worldwide, with a particularly significant burden among the older population (1). As the aging process accelerates in China, comprehensive health management for older CHD patients has become a major public-health issue. Among the various comorbidities that affect the prognosis of CHD, sleep disorders have garnered increasing clinical attention because of their high prevalence and often subtle presentation. Evidence-based research indicates that approximately 50–75% of patients with CHD experience varying degrees of sleep problems, which manifest in diverse forms, such as difficulty falling asleep, fragmented sleep, early awakening, and significantly reduced sleep efficiency (2). Notably, sleep disorders are not merely accompanying symptoms; they represent an independent risk factor that adversely affects recovery processes and long-term outcomes in patients with CHD. Large-scale cohort studies have confirmed that patients with CHD and coexisting sleep disorders face a significantly increased risk of malignant arrhythmias, recurrent myocardial infarction, and sudden cardiac death (3).

Older CHD patients constitute a highly vulnerable group for developing sleep disorders due to their unique physiological and pathological characteristics. On one hand, aging is associated with notable changes in sleep structure, such as decreased deep sleep and increased nighttime awakenings (4). In contrast, symptoms caused by myocardial ischemia (e.g., nocturnal chest discomfort) and heart failure-related conditions such as orthopnea or paroxysmal nocturnal dyspnea can directly disrupt both sleep continuity and quality (5, 6). Additionally, older patients frequently present with multiple chronic diseases, and medications used to treat these conditions (e.g., beta-blockers or diuretics) may also affect central nervous system function or urinary patterns, further exacerbating sleep disturbances (7, 8). However, current clinical practice still shows insufficient attention to sleep problems in older patients with CHD. There is a lack of efficient and precise screening tools for assessing the risk of sleep disorders, resulting in many high-risk individuals remaining undetected and without timely intervention.

Traditional risk assessment of sleep disorders mostly depends on classical statistical methods such as Logistic regression. Although such methods have the advantages of clear model structure and strong interpretability, they often face challenges in processing real-world medical data. Clinical data usually contain many nonlinear relationships, complex interactions between variables, and varying degrees of collinearity. However, traditional models have limited ability to capture such complex data structures (9), which may lead to suboptimal prediction performance and loss of key information. In recent years, machine learning (ML) algorithms have demonstrated significant advantages in the field of medical prediction. Random forest (RF) effectively reduces the risk of overfitting associated with a single decision tree by integrating many decision trees and can automatically evaluate feature importance (10). Gradient boosting machine (GBM), as another powerful ensemble algorithm, uses a sequential construction method to continuously correct prediction errors from previous iterations through optimization, and has shown superior performance in many medical prediction tasks (11, 12).

However, there is currently no systematic study comparing the performance of GBM, RF, and Logistic regression in predicting sleep disorders in older patients with CHD. Therefore, based on routinely collected clinical indicators, this study aims to construct three predictive models—GBM, RF, and Logistic regression—and to comprehensively evaluate and compare their performance using rigorous validation methods, to identify the optimal predictive tool. This study provides a scientific basis for the early identification of sleep disorder risk and targeted intervention in older patients with CHD

## Materials and methods

### Study subjects

This cross-sectional study continuously enrolled older ( ≥ 60 years old) hospitalized patients with CHD from the Department of Cardiology, The Second Affiliated Hospital of Hunan University of Chinese Medicine, between January 2023 and December 2024. The inclusion criteria were as follows: (1) meeting the diagnostic criteria for CAD (13), with symptoms including chest tightness or chest pain worsening after activity, confirmed by coronary angiography; (2) age ≥ 60 years; (3) no language or hearing impairments; and (4) a conscious state allowing cooperation in completing the questionnaire surveys. The exclusion criteria were as follows: (1) severe cognitive impairment or mental illness; (2) concurrent terminal illnesses (e.g., malignancies); (3) hematological or coagulation dysfunction; (4) substance dependence (including drugs, alcohol, or other psychoactive substances); and (5) hepatic or renal insufficiency.

In addition, to verify the external validity of the model, another 152 older CHD patients in our hospital from January 2025 to October 2025 were collected as the external validation cohort, and the inclusion and exclusion criteria for these patients were the same as those described above. This study was approved by the Ethics Committee of the Second Affiliated Hospital of Hunan University of Chinese Medicine (2025-KY-073-01). Written informed consent was waived by the Ethics Committee due to the retrospective, de-identified nature of the data and minimal risk to participants. The study followed the 1964 Helsinki declaration.

### Sleep disorder assessment and grouping

The Chinese version of the Pittsburgh Sleep Quality Index (PSQI) was used to evaluate patients’ overall sleep quality over the past month. The PSQI comprises seven dimensions, with total scores ranging from 0 to 21 points. A PSQI score > 7 was used as the criterion for identifying sleep disorders (14). Accordingly, participants were categorized into two groups: sleep disorder group (PSQI > 7) and non-sleep disorder group (PSQI ≤ 7).

### Collection of clinical relevant data

Potential predictor variables were collected through the electronic medical record system and standardized questionnaires, including demographics such as age, gender, and body mass index (BMI); type of coronary heart disease; NYHA functional classification; comorbidities (hypertension, diabetes, dyslipidemia, atrial fibrillation); disease duration; smoking history; alcohol consumption; and medication use. Additionally, the Generalized Anxiety Disorder-7 (GAD-7) (15) was used to assess anxiety symptoms. It consists of seven items, with a total score ranging from 0 to 21, where scores ≥ 5 indicate the presence of anxiety symptoms. Depression was evaluated using the nine-item Patient Health Questionnaire (PHQ-9) (16), which includes nine items with a total score ranging from 0 to 27, with scores ≥ 5 indicating depressive symptoms.

### Quality control

The survey content underwent repeated expert review to ensure consistency of project responses and standards. Trained nurses distributed and administered the questionnaires, guided the respondents in completing them, and verified their accuracy. Data were subsequently double-entered, and 5% of the data were randomly audited to ensure the reliability and validity of the findings obtained during the analysis phase.

### Statistical methods

SPSS 29.0 was used for data analysis. Continuous variables with a normal distribution are expressed as mean ± standard deviation (SD), and the independent samples *t*-test was used for comparison. Categorical variables are presented as percentages (%), and the χ^2^ test was used for comparison. The total sample (*n* = 338) was randomly divided into a training set (70%, *n* = 241) and an internal validation set (30%, *n* = 97).

Least absolute shrinkage and selection operator (LASSO) regression with 10-fold cross-validation was performed on the training set to screen the factors influencing sleep disorders in older patients with CHD, using the 1-standard error (1-SE) criterion for λ selection. Based on the variables selected by LASSO, three prediction models were constructed: Logistic regression, RF, and GBM. For the RF model, hyperparameters were set as follows: ntree = 500, mtry = 2 (the square root of the number of variables), and node size = 1 (default). No class weighting was applied. A fixed random seed (123) was used for reproducibility. For the GBM model, hyperparameters were set as follows: interaction.depth = 3, shrinkage = 0.05, n.minobsinnode = 10 (default), bag fraction = 0.5, and Bernoulli loss function. The optimal number of iterations was determined using five-fold cross-validation.

The area under the receiver operating characteristic (ROC) curve (AUC) was used to evaluate model discrimination, and calibration curves were used to assess the model calibration. The DeLong test was used to compare the differences in AUC between the models. A temporal validation cohort (*n* = 152, same hospital in a later time period) was used to validate the Logistic regression model. All tests were two-sided, with a significance level of α = 0.05. Model construction and validation were performed using R software (version 4.2.0) with the “gbm,” “rms,” “caret,” and “randomForest packages.”

## Results

### Demographic data and single-factor analysis of sleep disorders in older patients with CHD

Among the 338 older patients with CHD, 82 (24.26%) had sleep disorders and 256 (75.74%) did not. There were no significant differences in age, education level, BMI, hypertension, hyperlipidemia, atrial fibrillation, smoking, drinking, medication use, hsCRP, or LVEF between the two groups (*P* > 0.05). The proportions of female sex, CHD duration ≥ 3 years, diabetes mellitus, chronic gastritis, anxiety, and depression in the sleep disorder group were higher than those in the non-sleep disorder group (*P* < 0.05, Table 1).

**TABLE 1:** Demographic data and single factor analysis of sleep disorders in older CHD patients.

Factor	Sleep disorder group (*n* = 82)	Non-sleep disorder group (*n* = 256)	t/χ ^2^	*P*
Sex			6.798	0.009
Female	52 (63.41)	120 (46.88)		
Male	30 (36.59)	136 (53.12)		
Age (years)			0.008	0.927
<75	37 (45.12)	117 (45.70)		
≥ 75	45 (54.88)	139 (54.30)		
Level of education			0.175	0.676
High school and below	55 (67.07)	178 (69.53)		
Above high school	27 (32.93)	78 (30.47)		
Course of coronary heart disease (years)			7.365	0.007
<3	26 (31.70)	125 (48.83)		
≥ 3	56 (68.29)	131 (51.17)		
BMI (kg/m^2^)			0.115	0.735
<24	45 (54.88)	135 (52.73)		
≥ 24	37 (45.12)	121 (47.27)		
Diabetes			6.272	0.012
Yes	33 (40.24)	66 (25.78)		
No	49 (59.76)	190 (74.22)		
Hypertension			0.352	0.553
Yes	46 (56.10)	134 (52.34)		
No	36 (43.90)	122 (47.66)		
Hyperlipidemia			0.102	0.750
Yes	18 (21.95)	52 (20.31)		
No	64 (78.05)	204 (79.69)		
Atrial fibrillation			0.464	0.496
Yes	13 (15.85)	33 (12.89)		
No	69 (84.15)	223 (87.11)		
Chronic gastritis			13.422	<0.001
Yes	24 (29.27)	31 (12.11)		
No	58 (70.73)	225 (87.89)		
Smoking			0.835	0.361
Yes	24 (29.27)	62 (24.22)		
No	58 (70.73)	194 (75.78)		
Drinking			0.001	0.982
Yes	19 (12.17)	59 (23.05)		
No	63 (76.83)	197 (76.95)		
Medication			8.106	0.230
Aspirin	13 (15.85)	36 (14.06)		
P2Y12 inhibitors	15 (18.29)	51 (19.92)		
ACEI/ARB	15 (18.29)	41 (16.02)		
β-blockers	17 (20.73)	27 (10.55)		
CCB	9 (10.98)	43 (16.80)		
Statins	8 (9.76)	35 (13.67)		
Diuretics	5 (6.10)	23 (8.98)		
Combined anxiety			37.103	<0.001
Yes	44 (53.66)	49 (19.14)		
No	38 (46.34)	207 (80.89)		
Depression combined			36.263	<0.001
Yes	45 (54.88)	52 (20.31)		
No	37 (45.12)	204 (79.69)		
hsCRP (mg/L)			0.592	0.441
>10	23 (28.05)	61 (23.83)		
≤ 10	59 (71.95)	195 (76.17)		
LVEF (%)	57.32 ± 10.50	57.14 ± 10.11	0.140	0.889

### Variable screening based on LASSO regression analysis

**FIGURE 1:**
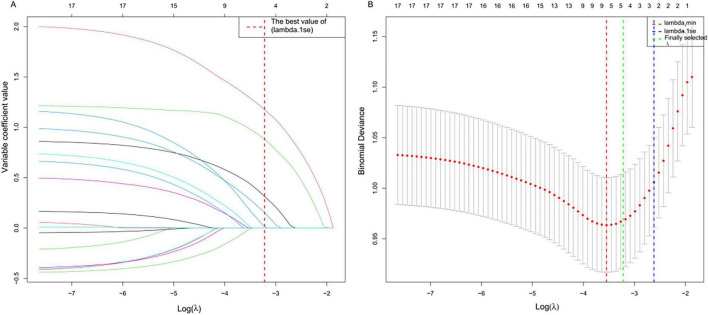
Variable screening based on LASSO regression analysis. **(A)** LASSO regression analysis variable screening dynamic diagram. **(B)** LASSO regression analysis 10-fold cross-validation plot.

Based on the training set data, 17 independent variables were included in the LASSO regression analysis to screen the risk factors affecting sleep disorders in older patients with CHD (Figure 1A). In the cross-validation curve, the left dotted line represents the optimal λ value of the evaluation index (Figure 1B), with eight variables having non-zero coefficients. The right dotted line represents the λ value within one standard error of the optimal value, with five variables having non-zero coefficients. The five potential variables were sex, duration of CHD, chronic gastritis, co-occurring anxiety, and co-occurring depression (Table 2).

**TABLE 2:** Variable screening of LASSO regression analysis.

Factors	λ +1se
Sex	0.314
The course of coronary heart disease	0.151
Chronic gastritis	0.017
Combined anxiety	1.176
Depression combined	0.881

### Establishment of the Logistic regression model

**FIGURE 2:**
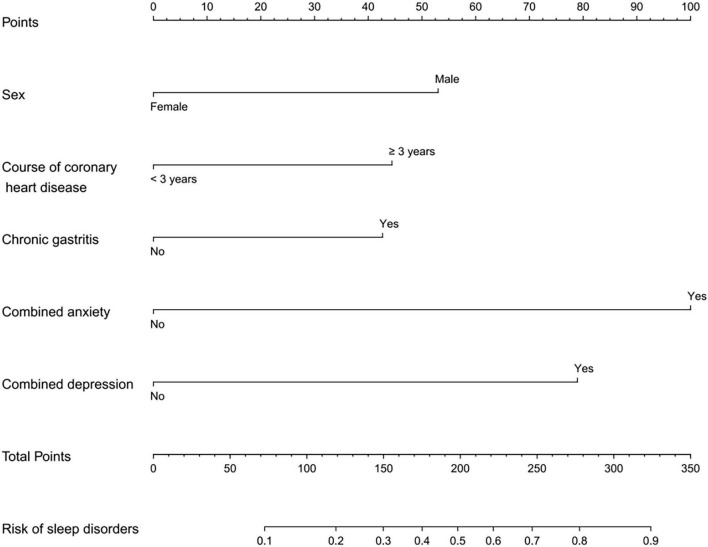
Logistic regression prediction model for the risk of sleep disorders in older patients with CHD. CHD, coronary heart disease.

Based on the five characteristic variables (sex, duration of CHD, chronic gastritis, co-occurrence of anxiety, and co-occurrence of depression) selected through LASSO regression, a nomogram prediction model for the risk of sleep disorders in older patients with CHD was constructed (Figure 2).

### Selection of characteristic variables and establishment of the GBM model

**FIGURE 3:**
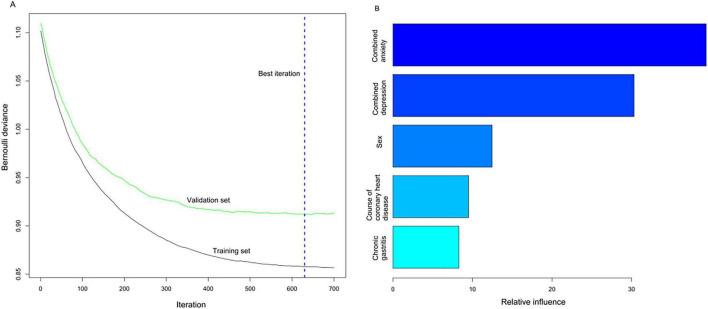
Establishment of GBM model. **(A)** The relative importance ranking diagram of variables in GBM model. **(B)** The relative importance of variables in the GBM model.

Based on the five risk factors selected by LASSO in the training set, a GBM model was established. Hyperparameter tuning was performed using five-fold cross-validation on the training set. The tuning included interaction.depth = 3, shrinkage = 0.05, and n.minobsinnode = 10 (default), with bag fraction = 0.5 and Bernoulli loss function. The optimal number of iterations was determined by cross-validation. When n.trees = 630, the model achieved the smallest generalization error (Figure 3A). The relative importance of clinical characteristics, ranked from highest to lowest, was as follows: co-occurring anxiety (39.39 points) > co-occurring depression (30.31 points) > sex (12.47 points) > duration of CHD (9.52 points) > chronic gastritis (8.31 points), as shown in Figure 3B.

### RF model establishment

**FIGURE 4:**
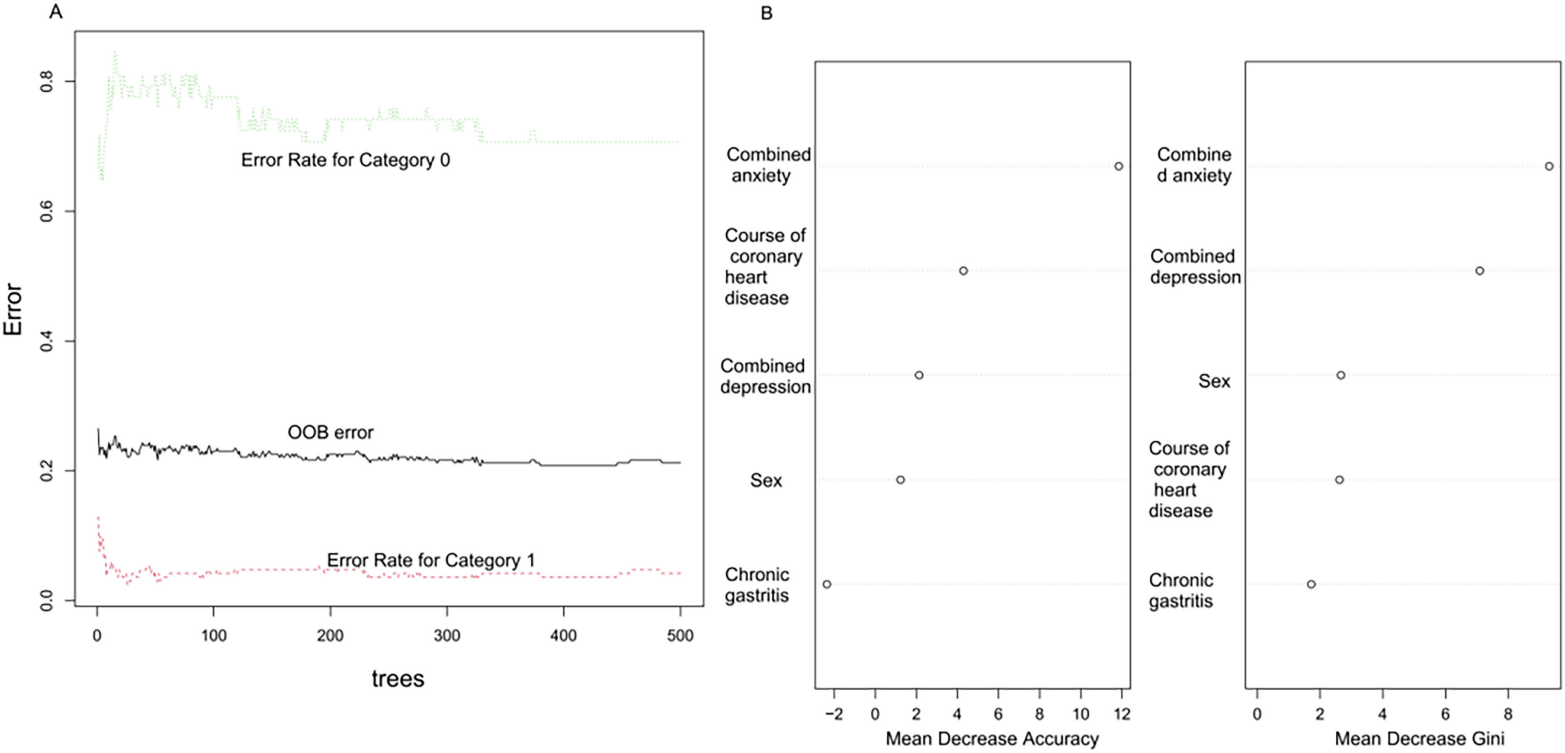
RF model establishment. **(A)** The dynamic relationship between the prediction error of the RF model and the number of random trees. **(B)** RF model variable Mean Decrease Gini importance ranking.

Based on the five risk factors selected by LASSO in the training set, an RF model was established. Hyperparameter tuning was performed using the default settings of the randomForest package. The final model used ntree = 500, mtry = 2 (floor of square root of 5 variables), and node size = 1 (default). No class weighting was applied. A fixed random seed (123) was used for reproducibility. At each node of the forest, the number of pre-selected variables was set as the square root of the total number of variables, and the random seed was set to 12,345. When ntree = 500 and mtry = 2, the error stabilized, indicating an optimal model. The dynamic relationship between the prediction error and number of trees is shown in Figure 4A. The importance of variables for predicting sleep disorders in older patients with CHD, ranked by the mean decrease in the Gini index, was as follows: co-occurring anxiety (9.30 points) > co-occurring depression (7.09 points) > sex (2.67 points) > duration of CHD (2.62 points) > chronic gastritis (1.72 points), as shown in Figure 4B.

### Comparison of predictive performance of the three models

**FIGURE 5:**
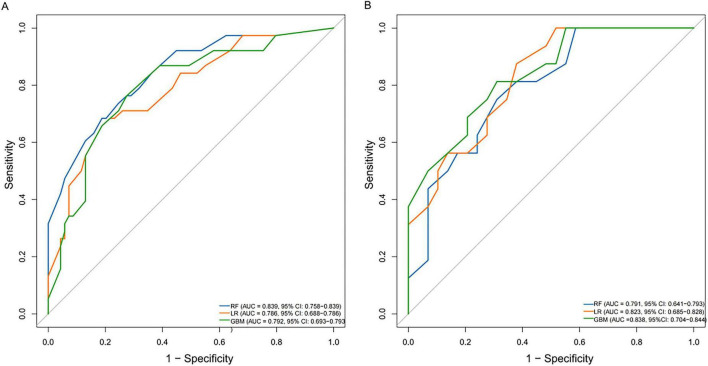
Three models’ ROC curves for predicting sleep disorders in older patients with CHD. **(A)** Training set. **(B)** Validation set. CHD, coronary heart disease; GBM, Gradient Boosting Machine; ROC, receiver operating characteristic.

**FIGURE 6:**
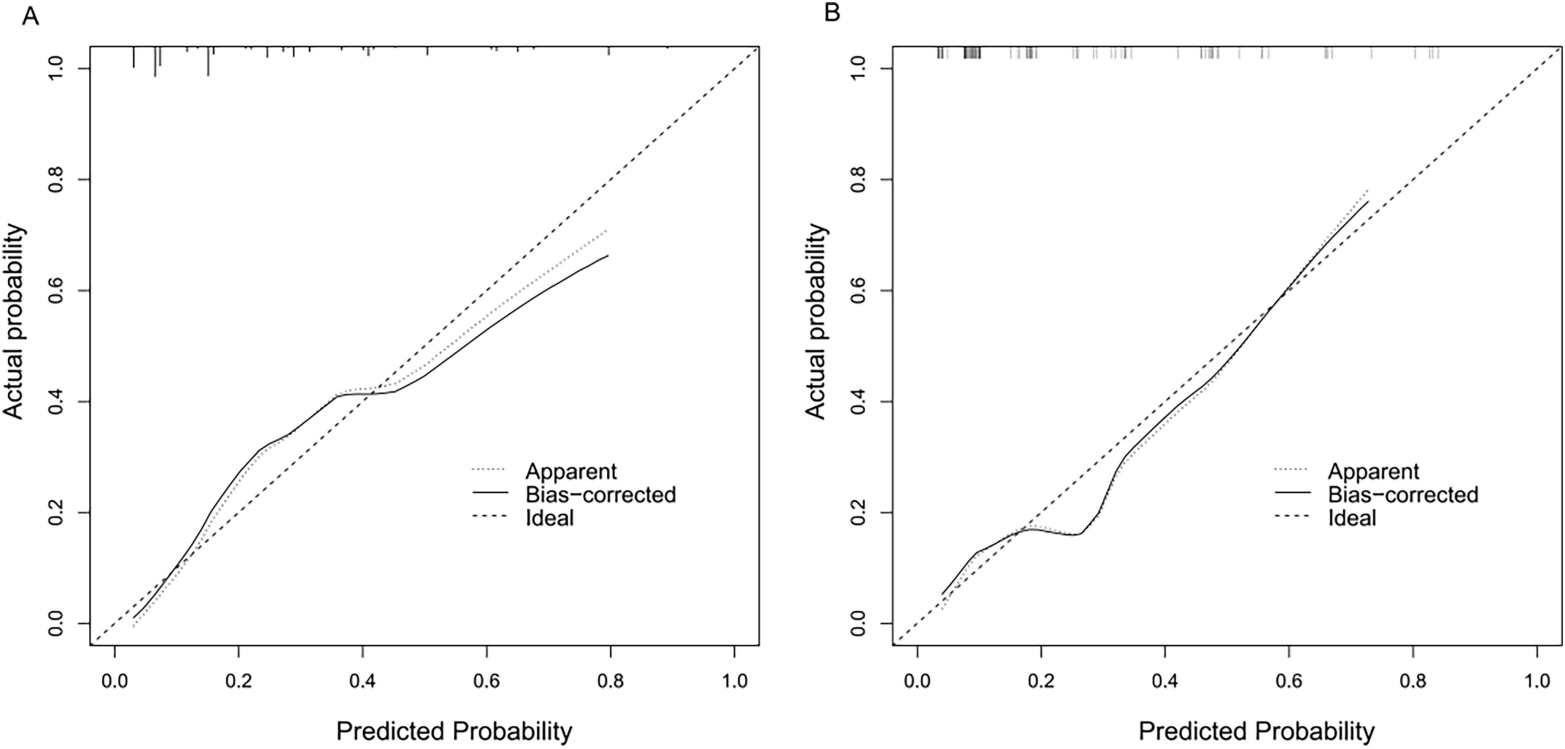
Calibration curve of the Logistic regression model for predicting sleep disorders in older patients with CHD. **(A)** Training set. **(B)** Validation set.

**FIGURE 7:**
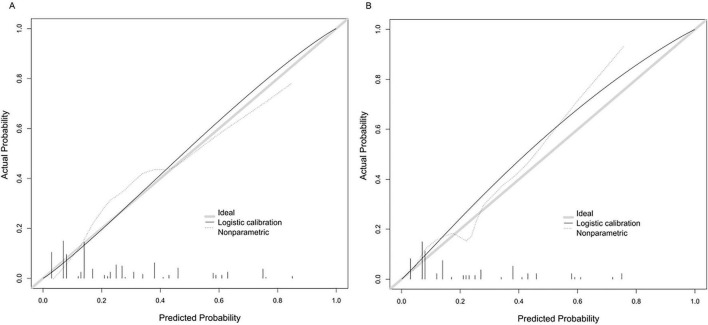
The calibration curve of the GBM model for predicting sleep disorders in older CHD patients. **(A)** Training set. **(B)** Validation set. CHD, Coronary heart disease; GBM, Gradient Boosting Machine.

**FIGURE 8:**
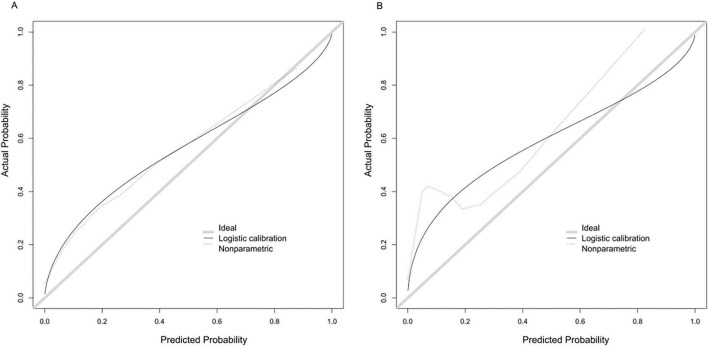
The calibration curve of RF model for predicting sleep disorders in older CHD patients. **(A)** Training set. **(B)** Validation set. CHD, coronary heart disease; RF, Random Forest.

The performance of the Logistic regression, GBM, and RF models was systematically compared in both the training and validation sets, and ROC curves and calibration curves were plotted for each model. In the training set, all three models demonstrated excellent predictive performance. The RF model had the highest AUC (0.839, 95% CI: 0.758–0.839), the highest accuracy (0.766), and the highest F1 score (0.818); the sensitivity of Logistic regression and the RF model was both 0.812; the specificity of the GBM model was the highest (0.763). In the validation set, the GBM model had the highest AUC (0.838, 95% CI: 0.704–0.844) and the highest accuracy rate (0.733); the Logistic regression model had the highest specificity (0.875) and the highest F1 score (0.825); the sensitivity of both the GBM and RF models was 0.690. The three models have good generalization capabilities. In summary, the RF model performs the best in terms of training set AUC and accuracy, the GBM model performs the best in validation set AUC, and the Logistic regression model performs the best in validation set specificity and F1 score (Table 3 and Figure 5). Confusion matrices of all models in the training and validation sets are provided in Table 4. The calibration curves indicate that the consistency between the predicted probabilities and the actual probabilities of the three models in the training set and validation set is good (Figures 6–8). The Delong test results showed that there was no statistically significant difference in the AUC values of the three models when compared pairwise (*P* > 0.05).

**TABLE 3:** Comparison of prediction performance of machine learning models.

Model	AUC (95%CI)	Sensitivity	Specificity	Accuracy	Precision	Recall Rate	F1	Optimal threshold
Modeling set								
Logistic regression	0.786 (0.688 ∼ 0.786)	0.757	0.812	0.658	0.812	0.812	0.812	0.393
GBM	0.792 (0.693 ∼ 0.793)	0.639	0.725	0.763	0.725	0.848	0.781	0.347
RF	0.839 (0.758 ∼ 0.839)	0.766	0.812	0.684	0.812	0.824	0.818	0.335
Validation set								
Logistic regression	0.823 (0.685 ∼ 0.828)	0.814	0.621	0.875	0.621	0.890	0.825	0.293
GBM	0.838 (0.704 ∼ 0.844)	0.711	0.690	0.813	0.690	0.870	0.769	0.348
RF	0.791 (0.641 ∼ 0.793)	0.711	0.690	0.750	0.690	0.833	0.755	0.296

RF, random forest; GBM, gradient boosting machine.

**TABLE 4:** Confusion matrices of the three machine learning models in the training and validation sets.

Model	True positive (TP)	False positive (FP)	True negative (TN)	False negative (FN)
Logistic regression				
Training	25	13	56	13
Validation	14	11	18	2
GBM				
Training	29	19	50	9
Validation	13	9	20	3
RF				
Training	26	13	56	12
Validation	13	9	20	4

### External validation

To evaluate model stability, a single-center temporal validation cohort of 152 older patients with CHD from our hospital (January 2025 to October 2025) was collected. The comparison of baseline characteristics between the external validation and training cohorts is shown in Table 5. There were no statistically significant differences in the clinical data between the two cohorts (*P* > 0.05).

**FIGURE 9:**
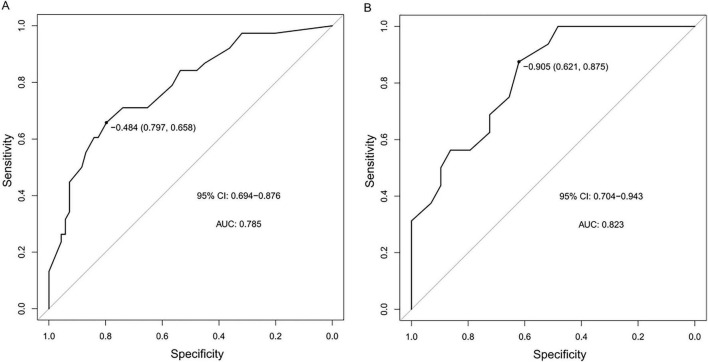
External validation of the ROC curve of the Logistic regression model for predicting sleep disorders in older patients with CHD. **(A)** Training set. **(B)** Validation set.

**FIGURE 10:**
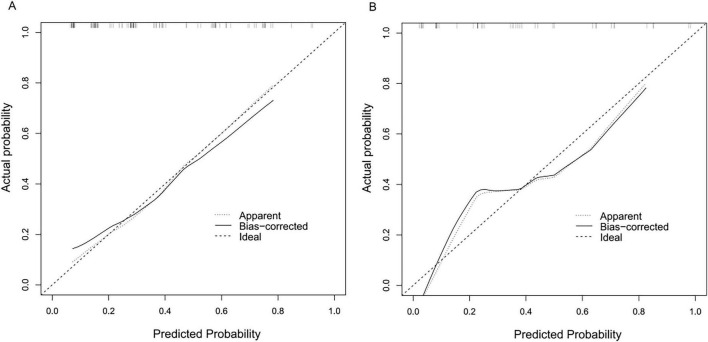
Calibration curve of the externally validated Logistic regression model for predicting sleep disorders in older patients with CHD. **(A)** Training set. **(B)** Validation set.

The predictive performance of the Logistic regression model was externally validated using the following methods. The results showed that in the training set, the AUC was 0.785 (95% CI: 0.694–0.876), with a sensitivity of 0.797 and a specificity of 0.658. In the validation set, the AUC was 0.823 (95% CI: 0.704–0.943), with a sensitivity of 0.621 and specificity of 0.875, indicating good discrimination (Figure 9). The calibration curve showed good agreement between the predicted and observed probabilities (Figure 10).

## Discussion

With the acceleration of population aging, the number of older patients with CHD continues to rise, and sleep disorders, as a common complication, have become an important factor affecting patients’ quality of life and prognosis. Sleep disorders are not only manifested as subjective symptoms such as difficulty falling asleep, sleep maintenance disorders, and early awakening, but may also further promote the progression of CHD by activating the sympathetic nervous system and aggravating oxidative stress and inflammatory responses, thereby forming a vicious cycle of “sleep disorders–CHD deterioration” (6, 17). This study found that among the 338 older patients with CHD included, the prevalence of sleep disorders was 24.26%, which is consistent with related reports (18). Notably, the incidence of sleep disorders in older patients is significantly higher than that in the general older population (approximately 15–20%), likely due to the combined effects of physiological decline (such as decreased melatonin secretion and circadian rhythm disorders) and comorbidities (such as diabetes and chronic kidney disease) (19).

In addition, the role of psychological factors in sleep disorders among older patients cannot be ignored. Studies have shown that approximately 40–60% of patients with CHD have comorbid anxiety or depression, which affects the function of the hypothalamic–pituitary–adrenal (HPA) axis, leading to increased cortisol levels and 5-hydroxytryptamine (5-HT) metabolic disturbances, thereby contributing to sleep problems such as difficulty falling asleep and early awakening (20, 21). Therefore, constructing a predictive model for sleep disorders in older patients with CHD is of great importance for breaking this vicious cycle and improving the long-term prognosis.

This study identified five independent influencing factors through LASSO regression. Among them, sex, duration of CHD, and symptoms of anxiety and depression showed results highly consistent with studies conducted in the past 5 years. However, chronic gastritis, as a newly identified factor, requires further investigation. Female patients had a higher risk of sleep disorders, which may be related to abnormal thermoregulation and social role stress caused by decreased estrogen levels in postmenopausal women. Estrogen deficiency may reduce melatonin secretion and shorten sleep cycles, while increasing the risk of anxiety, thereby forming a “hormone–sleep–psychology” interaction network (22–24).

In terms of psychological factors, the independent effects of anxiety and depression are consistent with the theory of psycho-cardiology, which suggests that cardiovascular diseases and psychological disorders share common neurobiological mechanisms (such as overactivation of the HPA axis and increased release of inflammatory factors). These findings highlight the importance of early screening and intervention using psychological assessment tools (such as the HADS) (25). Chronic gastritis may contribute to sleep disturbances through nocturnal gastric acid reflux, leading to symptoms such as cough and asphyxia (OR = 1.92, 95% CI: 1.15–3.21). Studies have shown that 64.3% of patients with obstructive sleep apnea have gastroesophageal reflux disease. Although reflux events do not directly alter AHI values, they may induce sleep fragmentation through mechanical stimulation or via vagal reflex pathways (26).

Furthermore, dysfunction of the brain–gut axis represents another potential mechanism. Inflammatory cytokines (IL-6, TNF-α) released during chronic gastritis may affect HPA axis function via vagal afferent pathways, disrupting cortisol rhythms and sleep architecture (27). Chronic gastritis may also affect sleep quality in CHD patients through *Helicobacter pylori* infection. This pathogen can increase cytokine and fibrinogen levels, while bacterial toxins may accelerate atherosclerosis progression, leading to vascular endothelial injury and an increased risk of CHD progression (28). Recent studies have further identified shared immunosenescence signatures between *H. pylori* infection and insomnia, involving MHC-II pathways and ceRNA networks (29). In addition, the duration of coronary heart disease was identified as an independent factor (OR = 1.08, 95% CI: 1.02–1.14), suggesting that long-term disease status may indirectly affect sleep quality through chronic pain, reduced mobility, and impaired social functioning. This mechanism requires further verification through longitudinal studies.

This study selected five influencing factors (sex, duration of CHD, chronic gastritis, co-occurring anxiety, and co-occurring depression) based on the LASSO regression analysis and constructed prediction models using Logistic regression, GBM, and RF algorithms. The results showed that in the training set, the AUC values of the Logistic regression, GBM, and RF models were 0.786, 0.792, and 0.839, respectively; in the validation set, the AUC values of the three models were 0.823, 0.838, and 0.791, respectively, indicating that all three models have good discriminative ability, which is consistent with the previous research results of machine learning models in the field of sleep and cardiovascular diseases (30). In the training set, the RF model had the highest AUC (0.839), and its accuracy (0.766), sensitivity (0.812), and F1 score (0.818) were all superior to the other models. In the validation set, the GBM model had the highest AUC (0.838), the highest accuracy (0.733); and the Logistic regression model had the highest specificity (0.875) and F1 score (0.825). Each of the three models had its own advantages in different indicators, and the overall performance was comparable, and all could be used as effective auxiliary tools for identifying elderly patients with coronary heart disease at high risk of sleep disorders. This is consistent with the recent data analysis results of NHANES, which also found that machine learning models (neural networks, random forests, XGBoost) performed well in predicting sleep disorders and cardiovascular disease risks (31).

**TABLE 5:** Comparison of clinical characteristics between external validation cohort and training cohort.

Factor	External validation queues (*n* = 152)	Training set queue (*n* = 241)	*t*/χ^2^	*P*
Sex			0.090	0.764
Female	79 (51.97)	129 (53.53)		
Male	73 (48.03)	112 (46.47)		
Age (years)			0.064	0.801
<75	63 (41.45)	103 (42.74)		
≥ 75	89 (58.55)	138 (57.26)		
Level of education			0.643	0.423
High school and below	104 (68.42)	174 (72.20)		
Above high school	48 (31.58)	67 (27.81)		
Course of coronary heart disease (years)			0.940	0.332
<3	77 (50.66)	110 (45.64)		
≥ 3	75 (49.34)	131 (54.36)		
BMI (kg/m^2^)			0.053	0.818
<24	82 (53.95)	128 (53.11)		
≥ 24	69 (45.39)	113 (46.89)		
Diabetes			0.001	0.971
Yes	42 (27.63)	67 (27.80)		
No	110 (72.37)	174 (72.20)		
Hypertension			0.420	0.517
Yes	75 (49.34)	127 (52.70)		
No	77 (50.66)	114 (47.30)		
Hyperlipidemia			0.146	0.703
Yes	34 (22.37)	50 (20.75)		
No	118 (77.63)	191 (79.25)		
Atrial fibrillation			0.010	0.920
Yes	22 (14.47)	34 (14.11)		
No	130 (85.53)	207 (85.89)		
Chronic gastritis			0.636	0.425
Yes	28 (18.42)	37 (15.35)		
No	124 (81.58)	204 (84.65)		
Smoking			1.255	0.263
Yes	47 (30.92)	62 (25.73)		
No	105 (69.08)	179 (74.27)		
Drinking			0.011	0.917
Yes	34 (22.37)	55 (22.82)		
No	118 (77.63)	186 (77.18)		
Medication			5.253	0.512
Aspirin	22 (14.47)	35 (14.52)		
P2Y12 inhibitors	19 (12.50)	47 (19.50)		
ACEI/ARB	23 (15.13)	41 (17.01)		
β-blockers	21 (13.82)	32 (13.28)		
CCB	24 (15.79)	36 (14.94)		
Statins	26 (17.11)	31 (12.86)		
Diuretics	17 (11.18)	19 (7.88)		
Combined anxiety			1.935	0.164
Yes	55 (36.18)	71 (29.46)		
No	97 (63.82)	170 (70.54)		
Depression combined			1.366	0.242
Yes	54 (35.53)	72 (29.88)		
No	98 (64.47)	169 (70.12)		
hsCRP (mg/L)			1.827	0.176
>10	48 (31.58)	61 (25.31)		
≤ 10	104 (68.42)	180 (74.69)		
LVEF (%)	56.88 ± 9.92	56.59 ± 10.26	0.281	0.779

It is worth noting that the RF model performed best in the training set (AUC 0.839), but its AUC in the validation set was 0.791, which was lower than that in the training set, suggesting a certain degree of overfitting. This might be related to the fact that the RF model builds multiple decision trees and learns the training data too thoroughly. In contrast, the GBM model had the highest AUC in the validation set (0.838), and the difference from the AUC in the training set (0.792) was small, indicating better generalization ability. The Logistic regression model had the highest specificity in the validation set (0.875), indicating its advantage in identifying non-sleep disorder patients and being suitable for clinical screening to exclude low-risk individuals. Study (32) pointed out that machine learning algorithms are driving the precise development of sleep disorder prediction, and optimizing feature selection can significantly improve model performance. The adaptability of the working principles of different models to the characteristics of the data may explain the above results. RF uses the Bagging integration method, by constructing a large number of mutually independent decision trees and voting to output the result, can effectively reduce the variance of the model, but is prone to overfitting on the training set. GBM uses the Boosting strategy, by serially iterating and optimizing to improve prediction accuracy, showing better generalization ability. Although Logistic regression is a traditional linear model, it had the highest specificity in the validation set in this study, still having clinical practical value. The calibration curve shows that the predicted probabilities of each model are highly consistent with the actual incidence rate, suggesting good clinical applicability. Through the Delong test comparison, there was no statistically significant difference in the predictive performance of the three models in the training set and validation set (*P* > 0.05), indicating that RF, GBM, and Logistic regression performed comparably in this prediction task. This study further verified the application value of machine learning models in sleep disorder risk prediction, corresponding to the recent research direction of basic models in the sleep field.

This study has several limitations. First, as a single-center cross-sectional study, the generalizability of the findings should be interpreted with caution, and further validation using multi-center prospective designs is required. Second, the assessment of sleep disorders relied on subjective scales and lacked objective sleep monitoring data; future studies should incorporate objective measures such as polysomnography. Third, potential confounding factors, such as social environment, were not included. Fourth, the prevalence of sleep disorders was 24.3%, resulting in class imbalance. Although Youden’s index was used to determine the optimal classification threshold to balance sensitivity and specificity, future studies with larger samples should explore balancing techniques such as class weighting or SMOTE. Fifth, the RF model showed a decrease in AUC from the training set (0.839) to the validation set (0.791), suggesting possible overfitting, which may be related to the relatively small validation sample size (*n* = 97); larger validation cohorts are needed for more stable estimates. In contrast, the GBM model demonstrated good generalization with comparable AUC between training (0.792) and validation (0.838) sets. Sixth, due to the cross-sectional design, causal relationships cannot be established; A potential bidirectional relationship between chronic gastritis and sleep disorders warrants further investigation through prospective studies. Seventh, the validation cohort was collected from the same hospital in a later time period, which should be more accurately described as temporal validation rather than true external validation; therefore, the generalizability of the findings to other populations requires further investigation. Eighth, only the Logistic regression model underwent temporal validation, as it was prioritized for its clinical interpretability and ease of implementation. The RF and GBM models, despite showing comparable predictive performance, were not temporally validated in this study. Future research should temporally validate all three models in independent multicenter cohorts and incorporate multi-omics data to enhance clinical applicability.

In summary, the prediction models based on sex, duration of CHD, chronic gastritis, anxiety, and depression demonstrated good discriminative ability and calibration. All three models are suitable for risk assessment and early screening of sleep disorders in older patients with CHD, with model selection depending on specific clinical needs.

## Data Availability

The original contributions presented in this study are included in this article, further inquiries can be directed to the corresponding author.
